# Altered Patterns of Phase Position Connectivity in Default Mode Subnetwork of Subjective Cognitive Decline and Amnestic Mild Cognitive Impairment

**DOI:** 10.3389/fnins.2020.00185

**Published:** 2020-03-20

**Authors:** Chunting Cai, Chenxi Huang, Chenhui Yang, Xiaodong Zhang, Yonghong Peng, Wenbing Zhao, Xin Hong, Fujia Ren, Dan Hong, Yutian Xiao, Jiqiang Yan

**Affiliations:** ^1^School of Informatics, Xiamen University, Xiamen, China; ^2^Department of Ultrasound, The First Affiliated Hospital of Xiamen University, Xiamen, China; ^3^Department of Computing and Mathematics, Manchester Metropolitan University, Manchester, United Kingdom; ^4^Department of Electrical Engineering and Computer Science, Cleveland State University, Cleveland, OH, United States

**Keywords:** subjective cognitive decline, amnestic mild cognitive impairment, default mode network, phase position connectivity, neuroimaging biomarkers

## Abstract

Alzheimer’s disease (AD), which most commonly occurs in the elder, is a chronic neurodegenerative disease with no agreed drugs or treatment protocols at present. Amnestic mild cognitive impairment (aMCI), earlier than AD onset and later than subjective cognitive decline (SCD) onset, has a serious probability of converting into AD. The SCD, which can last for decades, subjectively complains of decline impairment in memory. Distinct altered patterns of default mode network (DMN) subnetworks connected to the whole brain are perceived as prominent hallmarks of the early stages of AD. Nevertheless, the aberrant phase position connectivity (PPC) connected to the whole brain in DMN subnetworks remains unknown. Here, we hypothesized that there exist distinct variations of PPC in DMN subnetworks connected to the whole brain for patients with SCD and aMCI, which might be acted as discriminatory neuroimaging biomarkers. We recruited 27 healthy controls (HC), 20 SCD and 28 aMCI subjects, respectively, to explore aberrant patterns of PPC in DMN subnetworks connected to the whole brain. In anterior DMN (aDMN), SCD group exhibited aberrant PPC in the regions of right superior cerebellum lobule (SCL), right superior frontal gyrus of medial part (SFGMP), and left fusiform gyrus (FG) in comparison of HC group, by contrast, no prominent difference was found in aMCI group. It is important to note that aMCI group showed increased PPC in the right SFGMP in comparison with SCD group. For posterior DMN (pDMN), SCD group showed decreased PPC in the left superior parietal lobule (SPL) and right superior frontal gyrus (SFG) compared to HC group. It is noteworthy that aMCI group showed decreased PPC in the left middle frontal gyrus of orbital part (MFGOP) and right SFG compared to HC group, yet increased PPC was found in the left superior temporal gyrus of temporal pole (STGTP). Additionally, aMCI group exhibited decreased PPC in the left MFGOP compared to SCD group. Collectively, our results have shown that the aberrant regions of PPC observed in DMN are related to cognitive function, and it might also be served as impressible neuroimaging biomarkers for timely intervention before AD occurs.

## Introduction

Alzheimer’s disease (AD), which occurs more commonly in the elder, is a chronic neurodegenerative disease of impaired cognitive and memory (Ren et al., 2019; Wessels et al., 2019). Nevertheless, there are no agreed drugs or treatment protocols for patients with AD. Therefore, the timely detection and treatment of the early stages of AD is an urgent and realistic issue, which can improve symptoms of illness and alleviate the progression of the disease (Yang et al., 2019). Amnestic mild cognitive impairment (aMCI), as the phase close to AD, has a 10–15% probability of converting into AD per annum (Yang et al., 2017). Subjective cognitive decline (SCD) is referring to the fact that impaired cognition appealed by patients is entirely subjective without objective behavior of cognitive decline (Funaki et al., 2019), which has the certain likelihood of the development into aMCI and AD stages (Caillaud et al., 2019; Kim et al., 2020). Thus, from the above, we aim to adopt aMCI and SCD phases which may evolve into AD to explore the neural mechanism of the early stage of AD.

Resting-state functional magnetic resonance imaging (rs-fMRI), as one of the neuroimaging techniques, can offer a noninvasive method for the assessment of the cognitive mechanism of AD to a certain degree (Ferenci et al., 2002). Besides, in most studies of the early stage of AD based on rs-fMRI, the default mode network (DMN) has been emphasized highly for a long time (Banks et al., 2018). Anatomically, it includes the anterior DMN (aDMN) which is primarily composed of the ventromedial prefrontal cortex (vmPFC), and posterior DMN (pDMN) which mainly focuses on the posterior cingulate cortex (PCC) (Xu et al., 2016). Notice that the former mainly concerns memory extinction and self-referential mental idealization, while the latter is closely related to the function of episodic memory retrieval (Yang et al., 2017, 2018). Previously, it has been reported that amyloid deposits are detected with a great probability in the PCC which served as a primary part of DMN (Wang et al., 2013). The previous work has also indicated that the regions of DMN are abnormal and may serve as prominent hallmarks in the early stage of AD such as mild cognitive impairment (MCI) and SCD (Huang et al., 2018a; Scherr et al., 2019; Xie et al., 2019). More specifically, these disconnected areas that are connected from the DMN to the whole brain are considered to be associated with cognitive disorder (Huang et al., 2019; Zhao et al., 2020).

Hilbert transform (HT), which is characterized by rapidly and accurately describing the instantaneous position phase, is suitable for the analysis of non-stationary signals (Peng et al., 2005; Qian et al., 2015). A former study has suggested that frequency domain estimations such as the instantaneous phase position would provide a higher correlation between two signals than time-domain signals (Mandel and Atkins, 2016). Previous studies based on electroencephalography (EEG) have shown that prominently increased and decreased alpha spectral powers are found using HT in patients with AD in comparison with healthy controls (HC) (Babiloni et al., 2006; Fraga et al., 2013; Wang et al., 2020). Furthermore, it has been reported that time series from EEG was employed to obtain the altered instantaneous phase position of DMN using the HT method, which can be served as biomarkers (Thatcher et al., 2014; Wang et al., 2019). A previous investigation has also revealed that the brain activation patterns of DMN measured by HT were altered between the eye open and the closed eye (Wang et al., 2015). Besides, the former work on depressive disorder has indicated that the signals, extracted from the interesting regions of rs-fMRI, can reveal the aberrant brain regions using the HT method (Yu et al., 2018). Until now no experiments of HT in AD based on rs-fMRI, especially in DMN subnetworks, have been reported, which may be a new way to explore the neural mechanism of the early stage of AD.

Accordingly, our target aims to analyze the altered patterns of instantaneous phase position connectivity (PPC) in DMN subnetworks (include aDMN and pDMN networks) connected to the whole brain, and to explore whether there exists a relationship between the cognitive function and the aberrant regions. We hypothesized that there exist distinct variations of PPC in DMN subnetworks for patients SCD and aMCI, which might act as discriminatory neuroimaging biomarkers.

## Materials and Methods

### Participant

**FIGURE 1:**
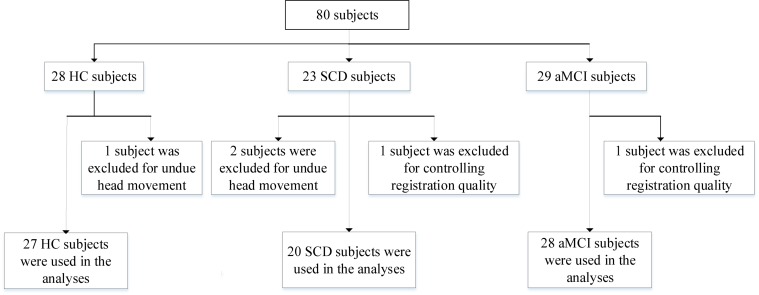
A flow chart depicting the data selection process. HC, healthy controls; SCD, subjective cognitive decline; aMCI, amnestic mild cognitive impairment.

Our experimental participants in this work are briefly summarized as follows: All subjects are obtained from the public database of the second phase of Alzheimer’s Disease Neuroimaging Initiative (ADNI-2)^1^. The emphasis of ADNI-2 is to survey neurological biomarkers of cognitive disorder. Furthermore, it was announced in 2011 which had been lasting for 5 years to implement. To further explore the gap between the healthy subjects and patients with MCI, consider joining the subjects with SCD for the first time in ADNI-2. All recruited subjects are consist of three parts, HC (*n* = 28), SCD (*n* = 23), and aMCI (*n* = 29), respectively. It deserves to be further mentioned that we have precluded three subjects as the result of undue head movement (cumulative translation or rotation >1.5 mm or 1.5 degrees were executed in our work, *n* = 3). At the same time, we strictly control registration quality relying on artificial visual recognition due to the poor registration of certain subjects (*n* = 2). To sum up, we recruited 75 subjects, including 27 HC, 20 SCD, and 28 aMCI participants. The detail data selection process is shown in Figure 1.

### MRI Data Acquisition

The obtained participants underwent rs-fMRI on a clinical 3.0 tesla (T) scanner (Philips Medical Systems). The echo-plane imaging (EPI) sequence consists of 140 volumes in which subjects were required to lie flat, close eyes, avoid conceptual work, and not turn the head during the process of data acquisition. Here, the specific parameters can be briefly described as: flip angle (FA) = 80 degree, matrix = 64 × 64, voxel size = 3.31 mm × 3.31 mm × 3.31 mm, repetition time (TR) = 3000 ms, echo time (TE) = 30 ms, slice thickness = 3.3 mm, respectively. T1-weighted structural MRI were acquired from magnetization-prepared rapid gradient-echo (MPRAGE) sequence (Chen et al., 2016), likewise, detailed parameters are represented as: matrix = 256 × 256, layer thickness = 1.2 mm, voxel size = 1 × 1 × 1.2 mm^3^, TR = 6.81 ms, FA = 9 degree, TE = 3.16 ms, respectively.

### Data Preprocessing

The data preprocessing with aspect to the rs-fMRI of this work adopted Resting-State fMRI Data Analysis Toolkit plus (RESTplus)^2^, which is based on MATLAB2012a^3^ and Statistical Parametric Mapping (SPM12)^4^. Briefly steps on data preprocessing involving as follows: We discarded the first 5 of 140 points in time for each subject as the result of the instability of MRI signal induced by machine or human. Then, the remaining volumes were calibrated for controlling the effects of between slices or between subject volumes. It is stressed that we have ruled out the data on the condition of cumulative translation more than 1.5 mm or angular motion more than 1.5 degrees. Next in the normalization process, registration from the original space to Montreal Neurological Institute (MNI) space using T1-weight volumes was implemented to reduce the diversities between various subjects. To directly circumvent the subject variations, the images generated above have been smoothed using a Gaussian kernel of 6 × 6 × 6 of full width at half maximum (FWHW). Following this, to eliminate the influence of nuisance variables (Fox et al., 2009; Huang et al., 2018b), comprising of six head motion parameters, global mean signal, white matter signal and cerebrospinal fluid signal, were ruled out, respectively. At last, subject volumes were filtered at 0.01–0.08 Hz due to noise interferences, which may be induced by heartbeat and breathing.

### Statistical Analysis

For this study, the variance (ANOVA) and the chi-square test within the Statistical Package for the Social Sciences (SPSS) software version 22.0 were conducted to determine whether there exist prominent differences (*p* < 0.05) as to the data of demographic and neurocognitive between the HC, SCD and aMCI groups.

To better illustrate the differences of PPC in aDMN and pDMN networks between HC, SCD, and aMCI groups, one-way ANOVA, which is integrated into the software of Data Processing and Analysis for Brain Imaging (DPABI)^5^, was adopted after controlling the gender and age. As mentioned in former literature, multiple comparisons at cluster level using non-parametric permutation test can availably control the false positive rate existing in statistics (Winkler et al., 2016), and 1000 permutation times and the prominent cluster size >30 voxels (810 mm^3^, *p* < 0.05) were performed in this work. We have employed the two-sample *T*-test for calculating the differences between two groups (i.e., SCD and HC groups, aMCI and HC groups, aMCI and SCD groups), and that mask used in two-sample *T*-test was derived from ANOVA analysis. Besides, according to the recent report, there have identified that the non-parametric permutation test with Threshold-Free Cluster Enhancement (TFCE) can effectively achieve a good balance between family wise error (FWE) rate and reliability (Chen et al., 2018). Therefore, permutation test associated with TFCE, all integrated into PLAM module of DPABI, were employed as the method of multiple comparisons of this work, and we set cluster size >10 voxels (270 mm^3^, *p* < 0.05) as a prominent cluster (Xue et al., 2019).

### PPC of Two Time Series Using Hilbert Transform

The HT can analyze nonlinear and non-stationary signals, and it is fully self-adaptive and suitable for mutation signals. Besides, it is characterized by rapidly and accurately describing the instantaneous phase position which was adopted to explore the altered patterns of PPC of DMN subnetworks in this work (Martis et al., 2012). Hence, the HT was adopted using complex demodulation to compute the instantaneous phase position difference between each pair of the time series. Among them, one series is extracted from aDMN (or pDMN), the other series is extracted from the voxel within the whole brain. The HT that we follow can be briefly described as follows: Given a time series name as *x*(*t*), and the HT later referred to as *H*(*t*), is shown in formula (1).

(1) $$H\,\left(t\right)=\frac{1}{\pi }\,\int_{-\infty }^{+\infty }\frac{x\,\left(\tau \right)}{\pi \,\left(t-\tau \right)}\,d\,\left(\tau \right)=x\,\left(t\right)*\frac{1}{\pi \,t}$$

We first take two time series which represent aDMN (or pDMN) signal and the voxel signal of the brain as inputs to the formula (1) respectively. Next, using analytic signal *Z*(*t*) of *x*(*t*) to generate information about the phase position and amplitude, as shown in formula (2). Therefore, two analytic signals of aDMN (or pDMN) and the voxel of the brain are obtained.

(2) $$Z\,\left(t\right)=x\,\left(t\right)+i\,H\,\left(t\right)=A\,\left(t\right)\,e^{-j\,\phi_{x}\,\left(t\right)}$$

where *A*(*t*) represents signal amplitude, and ϕ_*x*_(*t*) is the instantaneous phase position of the signal. Then, we obtain phase difference *D*(*t*) according to phase positions of two signals, consisting of ϕ_*x*1_(*t*) and ϕ_*x*2_(*t*), and it is shown in formula (3). Here, we set the phase lock ratio as *m* = *n* = 1, and it indicates that the increase phase of two phase position is consistent.

(3) $$D\,\left(t\right)=m\,\varphi_{x_{1}}\,\left(t\right)-n\,\varphi_{x_{2}}\,\left(t\right)$$

Phase synchronization exponent of two signals can be seen from the formula (4).

(4) $$\lambda =\left|{<e^{i\,D\,\left(t\right)}>}_{t}\right|=\sqrt{{<\cos D\,\left(t\right)>}_{t}^{2}+{<\sin D\,\left(t\right)>}_{t}^{2}}$$

Note that < ⋅ >_*t*_ is the average at each time point with values ranging from 0 to 1. Furthermore, λ = 0 indicates no phase synchronization of two signals, whereas λ = 1 shows phase synchronization of two signals. That is, the larger the value of λ, the stronger the synchronization between two signals. Moreover, the seed-based method was conducted to analyze the altered PPC of DMN subnetworks. To identify the seed regions about aDMN and pDMN, 10-mm spherical regions of interest in aDMN (MNI space: 0, 52, −6) and pDMN (MNI space: 0, −53, 26) were adopted in our work (Zhang and Raichle, 2010; Xue et al., 2019). Collectively, we applied phase position synchronization generated by HT to investigate the altered connectivity between the DMN subnetworks and the whole brain, and aim to explore the relationship between the aberrant regions and cognitive function.

## Results

### Demographic and Neurocognitive Characteristics

Here, the demographic and neurocognitive characteristics of experimental participants were described in Table 1. The prominent differences verified by ANOVA analysis were the Age (*F* = 8.248, *p* = 0.016), the MMSE score (*F* = 9.129, *p* < 0.01), and CDR score (*F* = 68.98, *p* < 0.01), respectively, yet no prominent difference on the Gender (*F* = 2.026, *p* > 0.05). The results showed that the MMSE values of the HC group (29.14 ± 1.49), SCD group (28.94 ± 0.83) and aMCI group (26.87 ± 2.72) decreased successively. It is noteworthy that lower MMSE scores suggest the severe form of cognitive impairment, whereas higher CDR scores show much more serious for dementia.

**TABLE 1:** Demographics and clinical measures of HC, SCD, and aMCI groups.

**Group**	**HC (*n* = 27)**	**SCD (*n* = 20)**	**aMCI (*n* = 28)**	***p*-values**
Gender	20F/7M	10F/10M	10F/18M	0.139^a^
Age (years)	72.63 ± 4.50	72.38 ± 5.31	69.71 ± 7.26	0.016^b^
MMSE scores	29.14 ± 1.49	28.94 ± 0.83	26.87 ± 2.72	<0.01^b^
CDR scores	0.03 ± 0.11	0.12 ± 0.22	0.52 ± 0.10	<0.01^b^

*Numbers are given as means ± standard deviation (SD) unless otherwise stated. MMSE, Mini-Mental State Examination; CDR, Clinical Dementia Rating; ^*a*^The *p*-values were obtained by the chi-square test. ^*b*^The *p-*value was obtained by one-way ANOVA analysis.*

### The Aberrant PPC of aDMN Network in Patients With SCD and aMCI

**FIGURE 2:**
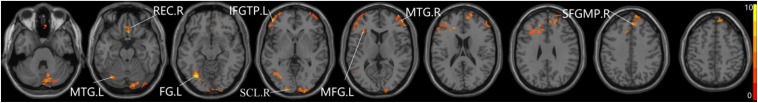
The prominent differences of the brain in PPC of the aDMN network using one-way ANOVA analysis. L, left; R, right; MTG, middle frontal gyrus; REC, rectus; FG, fusiform gyrus; IFGTP, inferior frontal gyrus of triangular part; SCL, superior cerebellum lobule; MFG, middle frontal gyrus; SFGMP, superior frontal gyrus of medial part.

**FIGURE 3:**
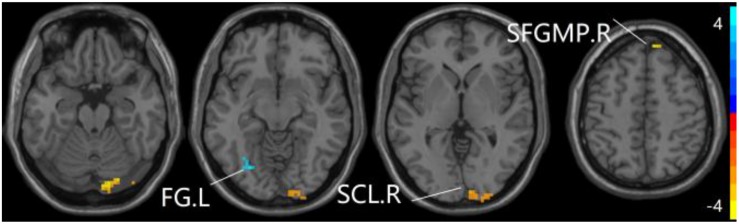
Compared to HC group, the SCD group exhibits prominent differences in PPC of the aDMN network based on two-sample *T*-test. L, left; R, right; FG, fusiform gyrus; SCL, superior cerebellum lobule; SFGMP, superior frontal gyrus of medial part.

**FIGURE 4:**
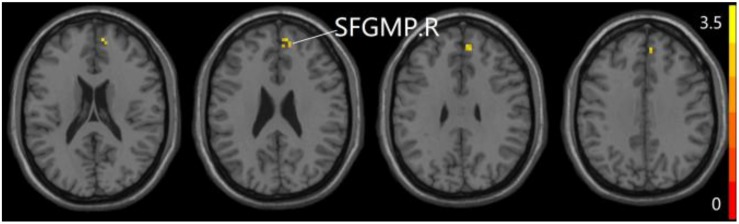
Compared to SCD group, the aMCI group exhibits prominent differences in PPC of the aDMN network based on two-sample *T*-test. R, right; SFGMP, superior frontal gyrus of medial part.

The studies we have performed indicated that eight prominent clusters, comprising of right superior cerebellum lobule (SCL), right rectus (REC), left fusiform gyrus (FG), left inferior frontal gyrus of triangular part (IFGTP), left middle temporal gyrus (MTG), right middle frontal gyrus (MFG), left MFG and right superior frontal gyrus of medial part (SFGMP), were revealed according to one-way ANOVA analysis. Besides, patients with SCD exhibited aberrant PPC in the clusters of right SCL, right SFGMP and left FG as compared with HC group, by contrast, no significant difference was found in patients with aMCI resulted from two-sample *T*-test. Notably, aside from decreased PPC found in the left FG, the clusters of increased PPC were involved in the right SCL and right SFGMP. It is important to note that patients with aMCI showed increased PPC in the right SFGMP in comparison with the SCD group (TFCE-FWE corrected, cluster size ≥ 10 voxels, *p* < 0.05). In particular, we emphasize that our experiments are after controlling the effects of age and gender (see Table 2 and Figures 2–4).

**TABLE 2:** The aberrant PPC in aDMN network.

**Region**	**Peak/MNI**			***T*-score**	**Cluster**
					**size**
	***x***	***y***	***z***		
**ANOVA**					
R Superior cerebellum lobule	−15	−99	−3	10.0818	323
R Rectus	3	33	−18	10.3223	45
L Fusiform gyrus	−30	−72	−12	9.5211	69
L Inferior frontal gyrus of triangular part	−48	51	3	9.9487	167
L Middle temporal gyrus	−51	−63	6	6.7590	37
R Middle frontal gyrus	54	39	15	10.7230	98
L Middle frontal gyrus	−36	27	12	7.8551	102
R Superior frontal gyrus of medial part	12	60	36	8.9812	204
**SCD > HC**					
R Superior cerebellum lobule	21	−93	−27	4.0315	227
R Superior frontal gyrus of medial part	12	48	51	3.9014	10
**HC > SCD**					
L Fusiform gyrus	−30	−72	−12	4.369	28
**aMCI > SCD**					
R Superior frontal gyrus of medial part	3	42	39	3.9141	24

*The *x*, *y*, *z* coordinates are the primary peak locations in the MNI space. Cluster size >30 voxels in one-way ANOVA analysis, *p* < 0.05. Cluster size >10 voxels in two-sample *T*-test, *p* < 0.05, TFCE-FWE corrected; L, left; R, right.*

### The Aberrant PPC of pDMN Network in Patients With SCD and aMCI

**FIGURE 5:**
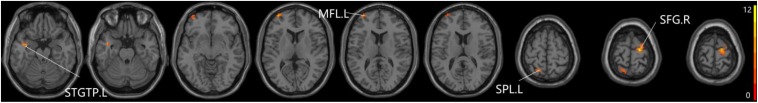
The prominent differences of the brain in PPC of the pDMN network based on one-way ANOVA analysis. L, left; R, right; STGTP, superior temporal gyrus of temporal pole; MFL, middle frontal lobule; SPL, superior parietal lobule; SFG, superior frontal gyrus.

**FIGURE 6:**
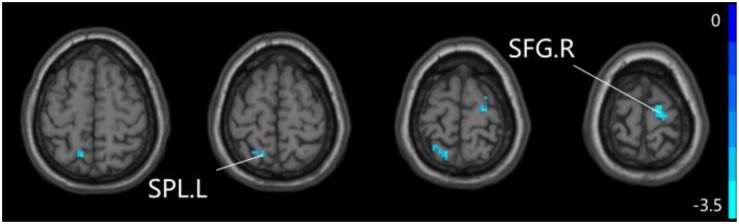
Compared to HC group, the SCD group exhibits prominent differences in PPC of the pDMN network based on two-sample *T*-test. L, left; R, right; SPL, superior parietal lobule; SFG, superior frontal gyrus.

**FIGURE 7:**
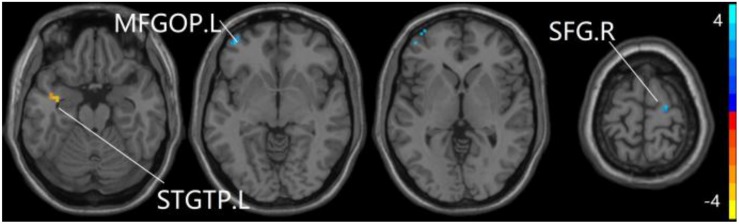
Compared to HC group, the aMCI group exhibits prominent differences in PPC of the pDMN network based on two-sample *T*-test. L, left; R, right; STGTP, superior temporal gyrus of temporal pole; SFG, superior frontal gyrus; MFGOP, middle frontal gyrus of orbital part.

**FIGURE 8:**
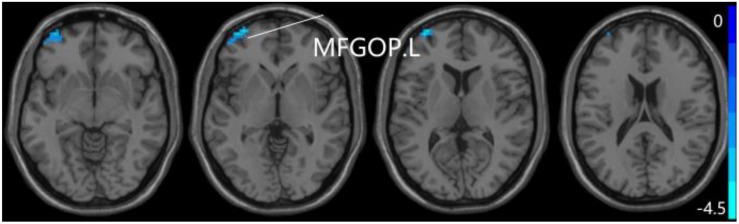
Compared to SCD group, the aMCI group exhibits prominent differences in PPC of the pDMN network based on two-sample *T*-test. L, left; MFGOP, middle frontal gyrus of orbital part.

For pDMN network, the one-way ANOVA analysis we have done suggested the prominent differences in four clusters, consisting of the left superior temporal gyrus of temporal pole (STGTP), left MFL, left superior parietal lobule (SPL) and right superior frontal gyrus (SFG). Compared to HC group, patients with SCD showed decreased PPC in the left SPL and right SFG. It is noteworthy that aMCI group showed decreased PPC in the left middle frontal gyrus of orbital part (MFGOP) and right SFG, yet increased PPC was found in the left STGTP. Additionally, aMCI group exhibited decreased PPC in the left MFGOP as the comparison with SCD group (TFCE-FWE corrected, cluster size ≥ 10 voxels, *p* < 0.05). The experimental data used in the PPC analysis are after controlling the influences of gender and age (see Table 3 and Figures 5–8).

**TABLE 3:** The aberrant PPC in pDMN network.

**Region**	**Peak/MNI**			***T*-score**	**Cluster**
					**size**
	***x***	***y***	***z***		
**ANOVA**					
L Superior temporal gyrus of temporal pole	−48	0	−15	11.6797	40
L Middle frontal lobule	−36	63	3	12.6064	94
L Superior parietal lobule	−15	−60	69	9.1601	32
R Superior frontal gyrus	18	−12	72	12.0896	52
**HC > SCD**					
L Superior parietal lobule	−15	−60	69	3.8077	25
R Superior frontal gyrus	12	−15	75	3.9821	36
**aMCI > HC**					
L Superior temporal gyrus of temporal pole	−48	0	−15	4.5391	31
**HC > aMCI**					
L Middle frontal gyrus of orbital part	−36	63	3	3.8683	16
R Superior frontal gyrus	18	−12	72	4.4364	18
**SCD > aMCI**					
L Middle frontal gyrus of orbital part	−36	63	3	4.7242	90

*The x, y, z coordinates are the primary peak locations in the MNI space. Cluster size > 30 voxels in one-way ANOVA analysis, *p* < 0.05; Cluster size > 10 voxels in two-sample *T*-test, *p* < 0.05, TFCE-FWE corrected; L, left; R, right.*

## Discussion

The work presented in this paper is that it focuses on investigating aberrant patterns of PPC of the DMN subnetworks connected to the whole brain between HC, SCD, and aMCI groups, and analyzing whether the aberrant areas are related with cognitive function. The result was consistent with the hypothesis that the SCD and aMCI groups exhibited distinct abnormal PCC in DMN subnetworks and the alerted regions were related to cognitive function. Besides, the altered regions in DMN subnetworks might regard as neuroimaging biomarkers and may be used to better understand the neural mechanism for the early stages of AD.

For aDMN, patients with SCD have prominently altered regions of the right SCL, right SFGMP and left FG resulted from the two-sample *T*-test. Based on earlier finds, SCL is involved in articulatory control and non-motor cognitive function (Ferenci et al., 2002). The left FG plays a critical role in semantic dementia and is repeatedly reported to be involved in visual word processing (Peterburs et al., 2019). SFG, which is located on top of the brain, plays a role in several higher-level cognitive processes and working memory according to a previous report (Alagapan et al., 2019). Combining the altered regions mentioned above, these regions are related to language and memory, which are consistent with the manifestation of cognitive function (Samaras et al., 2014). Interestingly, no prominent regions were found in aDMN network in aMCI group, while aMCI group exhibited prominent regions in left STGTP, left MFGOP and right SFG in pDMN. A former study has reported that STG is anchored in the auditory association cortex involving spoken word recognition and MFG is related to working memory (Fegen et al., 2015; Kajikawa et al., 2015). Hence, the altered regions we found might involve in language cognitive disorder, and pDMN first appears abnormal connections compared to aDMN, which might suggest that the aDMN and pDMN have different manifestations in the early stages of AD.

We found that both aMCI and SCD groups showed a prominent region in SFG that is connected to DMN subnetworks, and according to the previous research that the altered functional connectivity between the pDMN and the SFG might be a compensatory response of brain (Xue et al., 2019). We assume that the aberrant PPC in SFG connected to the DMN may also be caused by the brain’s compensation. Compared to HC group, aMCI group showed a prominent region in STG which did not belong to the altered region of SCD group, while SCD group showed prominent regions in SCL which did not belong to the altered region of aMCI group. It can be deduced that SCL and STG, related to articulatory control and working memory, respectively, are sensitive and might as neuroimaging biomarkers to distinguish the SCD and aMCI. Interestingly, compared to patients with SCD, DMN subnetworks in aMCI group showed obvious differences in right SFGMP and left MFGOP belonging to FG. In previous studies, aMCI group showed more widespread topological changes involving the frontal lobes (Barban et al., 2017), and aberrant connectivity was also found in patients with SCD between DMN and FG due to cognitive impairment (Xue et al., 2019). Besides, an increasing trend of values of amplitude of low-frequency fluctuation (ALFF) and fractional ALFF were detected in FG (Yang et al., 2018). Therefore, changes in FG may be related to cognitive dysfunction.

## Conclusion

Our study mainly shows that the PPC of the DMN subnetworks which are connected to the whole brain has different disconnection patterns in SCD and aMCI stages. Moreover, the significant difference in DMN subnetworks varies considerably, which might act as neuroimaging biomarkers of sensitivity for timely detection of the early stage of AD.

## Data Availability Statement

Publicly available datasets were analyzed in this study. This data can be found here: http://adni.loni.usc.edu/.

## Author Contributions

CC was responsible for writing the manuscript and doing the experiment. CH, CY, and XZ instructed the experiment. WZ, XH, FR, DH, YX, and JY participated in the experiment of manuscript and checked the English grammar. YP developed the idea for the study and analyzed most of the data.

## Conflict of Interest

The authors declare that the research was conducted in the absence of any commercial or financial relationships that could be construed as a potential conflict of interest.

## Footnotes

1 http://adni.loni.usc.edu/

2 http://restfmri.net/forum/RESTplusV1.2

3 http://www.mathworks.com/products/matlab/

4 https://www.fil.ion.ucl.ac.uk/spm/

5 http://rfmri.org/dpabi
